# The effect of kisspeptin on the maturation of human ovarian follicles in culture following vitrification-thawing processes

**DOI:** 10.5935/1518-0557.20230045

**Published:** 2023

**Authors:** Anahita Tavakoli, Ali Tayyebi Azar, Neda Taghizabet, Fatemeh Rezaei-Tazangi, Shahla Noori Ardebili, Zahra Shams Mofarahe, Fereshteh Aliakbari, Malek Soleimani Mehranjani

**Affiliations:** 1 Men’s Health and Reproductive Health Research Center, Shahid Beheshti University of Medical Sciences, Tehran, Iran; 2 Department of Biology, Faculty of Science, Arak University, Arak, Iran; 3 Clinical Research Development Unit of Imam Khomeini Hospital, Urmia University of Medical Sciences, Urmia, Iran; 4 Department of Anatomy, School of Medicine, Fasa University of Medical Sciences, Fasa, Iran; 5 Department of obstetrics and gynecology, Atieh hospital, Tehran, Iran; 6 Department of Biology and Anatomical Sciences, School of Medicine, Shahid Beheshti University of Medical Sciences, Tehran, Iran

**Keywords:** BMP15, follicle maturation, GDF 9, human ovarian cryopreservation, kisspeptin

## Abstract

**Objective:**

Ovarian cryopreservation is one of the effective methods to preserve fertility for cancer patients. Still, this approach has some problems, namely ROS, resulting in adverse effects on oocytes and ovarian follicles. Kisspeptin as an antioxidant to control ovarian function, directly or indirectly. In this study, the effect of kisspeptin on follicle maturation was evaluated in culture following ovarian cryopreservation.

**Methods:**

Ovarian tissue samples of women between 20 and 35 years old (n=12) were laparoscopically collected. The samples were randomly divided into four groups: 1) control, 2) vitrification, 3) vitrified+1µM kisspeptin, and 4) vitrified+10µM kisspeptin. After vitrification and thawing processes, the tissues were cultured in DMEM medium for 7 days. H&E staining for histological evaluation, Real-Time PCR for GDF9 and BMP15 gene expression, and immunohistochemical staining for GDF9 and BMP15 protein expression were performed.

**Results:**

In the vitrification group, ovarian tissue morphology was incoherent, and more primordial follicles than other follicle types were found. The expression of GDF9 and BMP15 genes and proteins were significantly decreased in this group compared with other groups (*p*<0.05). In the vitrification groups with kisspeptin (1 and 10 µM), the number of primary and secondary follicles was more than in the vitrification group. Besides, the expression of these genes and proteins was dramatically elevated in the vitrification groups with kisspeptin compared to the vitrification group alone (*p*<0.05).

**Conclusions:**

It seems that kisspeptin is an effective substance to improve the quality of the human ovarian cryopreservation medium by improving follicle maturation.

## INTRODUCTION

Yearly, a great number of reproductive-age adult women all over the world find out they have cancer. One of the main challenges for using common medical approaches for cancer treatment in women is maintaining women’s fertility, especially in underage girls (Rivas Leonel *et al*., 2019). Treatment methods for cancer, such as radiation therapy and chemotherapy, increase the survival rates of patients; however, these treatments can lead to POI (Primary ovarian insufficiency) and eventually pregnancy disruption (Blumenfeld, 2012; Kim *et al.*, 2021). Even demands for fertility preservation for non-oncological reasons are increasing day by day (Donnez & Dolmans, 2017). Thus, finding effective techniques for fertility preservation in these cases is essential (Kim *et al*., 2021; Aliakbari *et al*., 2022).

Among this, ovarian cryopreservation has been introduced as a useful method for prepubertal girls with cancer, and patients who are unable to delay treatment or ovarian stimulation is harmful to them (Jadoul *et al*., 2010; Wallace *et al*., 2014). Recently, cryopreservation of ovarian tissue by vitrification has been considered an important tool for the preservation of female fertility, in particular for subjects undergoing chemotherapy (Yang *et al*., 2016). Vitrification is a more cost-effective and faster method than slow freezing and it also prevents the formation of ice crystals in cells, causing high rates of embryo development, cell survival, and fertilization (Shi *et al*., 2017; Huo *et al*., 2021). Also, thawing is known as a fast way to prevent the re-crystallization process in which water hits embryos or oocytes and can be transformed into a solid form around, creating small ice crystals (Chian *et al*., 2014). One of the main results of the vitrification and thawing processes is ROS (Reactive oxygen species) formation through various mechanisms, like osmotic stress, oxidative metabolism, and changes in cell defense mechanisms that can lead to apoptosis stimulation and subsequent DNA damage (Mazoochi *et al*., 2009; Tatone *et al.*, 2010; Rocha *et al*., 2018).

In the women’s reproductive system, some agents suppress the apoptosis of granulosa cells and follicular atresia and stimulate granulosa cell mitosis, e.g., *BMP15 (*Bone morphogenetic protein 15) and *GDF9 (*Growth differentiation factor 9) (Gode *et al*., 2011). Several studies have been performed to decrease cryopreservation-stimulated cell injuries; however, there is no efficient method to date to treat these lesions (Rocha *et al*., 2018). In this way, kisspeptin (metastin), as a neuropeptide whose gene (*KISS1*) is located on chromosome 1q32.11, regulates the expression of antioxidant enzymes against oxidant agents and controls puberty and reproductive activities through its membrane receptors coupled with the G protein (Kotani *et al*., 2001). Kisspeptin triggers hypothalamic pituitary gonadal axis inducting gametogenesis by releasing FSH (Follicle stimulating hormone) and LH (Luteinizing hormone) through the pituitary gland (Aslan *et al*., 2017; MacManes *et al*., 2017). Also, reports expressed the role of kisspeptin in ovarian function control, such as follicular development, steroidogenesis, oocyte maturation, and ovulation (Hu *et al*., 2018). Despite these, according to our knowledge, the effectiveness of this neuropeptide on the maturity of human ovarian follicles in a vitrification medium has not been investigated. Hence, in this investigation, we studied the effect of kisspeptin in culture following vitrification-thawing methods on follicle maturation and the expression of related genes and proteins (*BMP15* and *GDF9*) in vitro for the first time.

## MATERIALS AND METHODS

### Sample collection

This study has been approved by the Shahid Beheshti University of Medical Sciences (ethical code: IR.SBMU.RETECH.REC.1399.554). We used the ovarian tissues of 20 women between the ages of 20 and 35 who underwent a hysterectomy and had their fallopian tubes ligated. All the patients were informed about the study, and their consent was obtained. The health of human ovarian tissue was examined by an obstetrician, and only women with normal BMI (Body mass index) (<27kg/m^2^) (Diamanti-Kandarakis & Bergiele, 2001) and normal AMH levels (Anti-mullerian hormone) (1.66ng/ml) (Yang *et al*., 2017) were included in the project (Table 1). Exclusion criteria were: Ovaries damaged for any reason during surgery, etc., as well as cancerous ovaries, ovaries without normal follicles, ovaries of people undergoing chemotherapy or hormone therapy, ovaries of people with addiction, ovaries of people using corticosteroids, and polycystic ovaries (Hardy, 2018). A sample of each ovary was taken to check the quality of follicles histologically by H&E (Hematoxylin and eosin) staining. Finally, 12 ovaries were healthy and had normal follicles, and others were excluded. Table 1 depicts the characteristics of the subjects involved in the study are shown in Table 1.

**Table 1:** Sample donors characteristics.

Samples	Age	Marital status	Age of first period	AMH	BMI
1	28	Single	11	2/5	22.65
2	27	Single	11	2/4	16.29
3	32	Married	14	7/29	20.95
4	32	Single	15	2	18.25
5	26	Single	10	4/12	26.81
6	38	Married	12	4/9	24/09
7	30	Single	13	7/3	21.93
8	24	Single	13	2/3	21.56
9	34	Married	11	16/6	24.45
10	27	Married	14	2/2	19.33
11	28	Married	13	14/2	21.36
12	30	Married	11	3/57	22.21

The tissues of those ovaries removed laparoscopically by the obstetrician were transferred to the laboratory in 10% HAMS (Ham’s tissue culture medium) solution and 20% human albumin serum with ice at -4°C for one h. The ovarian tissues were divided into 5 x 5 x 1 mm pieces with a sharp blade. All steps were performed on ice and under the laminar hood. Human ovarian cortex tissue parts were randomly allocated into four groups. All the mentioned chemicals were purchased from Sigma-Aldrich Chemie, Steinheim, Germany.

### Experimental groups

From each one of 12 ovaries obtained we collected 12 samples. We assigned three ovarian samples from each one of 12 samples to each group and, eventually, each of the experimental groups was further divided into four groups:

- Control group: Fresh ovarian samples (n=36)
- Vitrification group: Ovarian samples underwent culture following vitrificationthawing methods (without adding Kisspeptin) (n=36)
- Vitrification with + 1 µM kisspeptin group: 1 µM kisspeptin was added to vitrification medium and ovarian samples underwent vitrification-thawing methods (n=36)
- Vitrification + 10 µM kisspeptin group: 10 µM kisspeptin was added to vitrification medium and ovarian samples underwent vitrification-thawing methods (n=36)

### Vitrification

The ovarian tissue samples were placed by a carrier for 25 min in an ES (Equilibration solution) medium containing 7.5% ethylene glycol and 7.5% DMSO (Dimethyl sulphoxide) in 10% HAMS as a handling medium and then followed by a second equilibration VS (Vitrification solution) for 15 min containing 20% ethylene glycol, 20% DMSO, and 0.5 mol/ L Sucrose with 10% HEPES (4-(2-hydroxyethyl)-1-piperazineethanesulfonic acid). Kisspeptin was added to the VS medium at the concentration of 1 µM for group III and 10 µM for group IV.

### Thawing

For the thawing process, the ovarian samples were first immersed in a T₁ medium containing 1 mole sucrose and 10% HEPES with HAMS as the base medium for 1 min and then for 5 min in a T₂ medium containing 10% HEPES and 0.5 mol sucrose followed by putting them in a T₃ medium containing 10% HEPES and 0.25 mol sucrose for 10 min.

### Tissue culture

After thawing, the ovarian samples were cultured for seven days. The medium was DMEM 12 (Dulbecco’s modified eagle medium) ready culture with 10% FBS (Fetal bovine serum), and 5% Streptomycin and Penicillin antibiotics were used in the incubator to maintain the tissue for different stages of the tests. The culture medium was changed every two days and it didn’t contain kisspeptin.

### Histological assessments

All tissue samples after vitrification-thawing processes were fixed in 10% formalin. Tissue samples were embedded in paraffin blocks after tissue processing through graded alcohol and xylene solutions. Paraffin blocks were serially sectioned at a thickness of 5 µm. Afterwards, the sections were stained with H&E, mounted, and observed under light microscopy (Olympus, Tokyo, Japan). The identification and classification of follicles were done according to the study by Dehghani *et al*. (2018).

### Quantitative real-time polymerase chain reaction

In all groups, 2 or 3 fragments were homogenized and total RNA was extracted (Parstous, Iran). Then, isolated RNAs were converted to cDNA by cDNA synthesis kit (Parstous, Iran). Appropriate primers for *BMP15, GDF9*, and *GAPDH* genes were designed using Gene Bank database (http://www.ncbi.nlm.nih.gov) and primer software (Blast, MEDUSA, Primer 3 and UCSC). The primer sequences, product length, and GenBank access numbers are shown on Table 2. The primers were ordered from and synthesized at Sinaclon Company, Iran. In this investigation, the master mix PCR from (Parstous, Iran) was used comprising 5µl 2χ Master Mix, 0.5 µl Forward primer (10 µM), 0.5 µl Reverse primer (10µM), and 3µl H₂O (Total Volume=9µl + 1µl cDNA). All PCR materials were prepared in a 0.2 ml microtube and mixed and spun. The final mixture was distributed in the volumes of 9 µl in PCR microtubes, and 1 µl cDNA or DNA was added to each microtube. Next, the microtubes were put in the Real-time PCR thermocycler (ABI Step One, USA) at 95 °C for 5 min (Initial denaturation), 95°C for 15 sec, 50-60°C for 20 sec, 72°C for 30 sec (Amplification) and 4°C for 5 min (cooling). To check the gene expression and the specificity of PCR products, agarose gel with a suitable percentage depending on the length of the PCR product was prepared, and all PCR products were electrophoresed.

**Table 2:** Sequence characteristics of primers used to measure the expression of GDF9 and BMP15 genes.

Gene	Primer (5´- 3´)	Product length(bp)	Annealing temperature(˚C)	GenBank accession number
h_BMP15	GTGGTGGTCTTGAGCTCTGG(Forward) CATCTGCTTGTCGGGTTCTC(Reverse)	168 bp	60 58	NM_005448.2
h_GDF9	GGCACGTACACATGACGGTCT(Forward) CGCAGAGGTCAGGAAACTGTC(Reverse)	315 bp	62 61	NM_005260.7
h_GAPDH	CTTTGGTATCGTGGAAGGAC(Forward) GCAGGGATGATGTTCTGG (Reverse)	126 bp	56 55	NM_001357943.2

### Preparation of the electrophoresis gel

**Figure 1:**
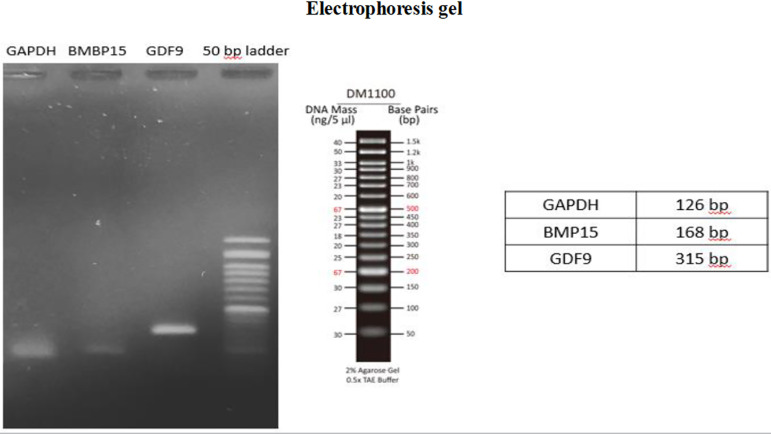
(A) shows ovarian tissue in the control group. In this group, more coherence was seen in the tissue than in other groups. (B) shows the H&E staining in the vitrification group and the primordial follicles can be seen. Also, tissue cohesion due to the vitrification and staining steps is less than in the control group. (C) indicates tissue incoherence, and primary follicles were seen in this group. (D) reveals the incoherence of primary and secondary tissues and Graafian follicles.

To prepare the 1% gel, 0.5 g agarose was mixed with 10 ml TBE buffer (Merck, Germany) in an Erlenmeyer flask, which was placed on heat until it became clear and reached a temperature of 70 degrees Celsius. Next, the special dye (safe stain Merck, Germany) was added to the mixture and poured into the electrophoresis tank. The gel was covered with 1% buffer. After the gel hardened, 3 µl of the product was combined with 1 µl buffer containing the dye (Merck, Germany) and inserted into the electrophoresis tank wells. A sizing solution (Sigma Aldrich, Germany) was poured into one of the wells, and the length of parts was determined accordingly. After entering the samples, the electrophoresis device was set to a voltage of 80 and a time of 30 min. After that, the gel was transferred to the screen of the trans luminaire device (Royan, Iran), and the band of samples was analyzed for the presence of the band, density, and dimensions (Figure 1).

### Immunohistochemistry

For IHC staining, paraffin-embedded ovarian tissue slides were deparaffinized with xylene and rehydrated with a series of ethanol solutions (50%, 70%, 80%, 90%, and 100%). 2 N hydrochloric acid was poured on the samples for 30 min, then borate buffer was added for 5 min. Tissue samples were washed with phosphate-buffered saline (PBS), and then 0.3% Triton was used for 30 min to permeate the cell membranes and washed with PBS. Afterward, 10% goat serum was added for 30 min as an additional background color. The primary antibody (GDF9-orb13431 and BMP15-orb247897) (1: 100) was diluted with PBS and added to the samples, then placed in a refrigerator at 2 to 8°C overnight after creating a humid environment to prevent tissue drying. After 24 h, the tissue container was removed from the refrigerator and then washed 4 times with PBS for 5 min each time. Secondary antibodies (orb688925) were added to the samples at a dilution of 1 to 150 and subsequently incubated at 37°C for 1 h and 30 min in the dark. The samples were transferred from the incubator to a dark room, and after 4 washings, DAPI (4′,6-diamidino-2-phenylindole) was added to them, immediately removed, and poured on the PBS samples. In the last step, the samples were studied in an Olympus fluorescent microscope (Olympus, Tokyo, Japan) (400 X) to confirm the markers.

### Statistical analyses

The data were analyzed statistically by the GraphPad Prism software, version 8.4.3, using the one-way ANOVA for the differences between more than two groups and Tukey’s tests for differences between groups. The results were reported as mean ± SEM. The level of significance was considered at *p*<0.05.

## RESULTS

### Histological findings

**Figure 2:**
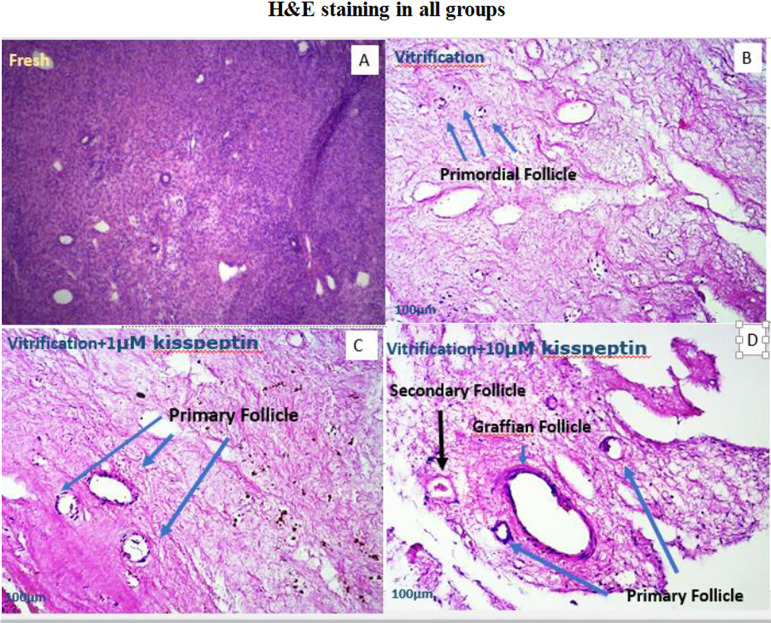
Gel electrophoresis of GDF9, BMP15, and GAPDH genes.

In the qualitative investigation of human ovarian tissue, there was more tissue cohesion in the control group than in the other groups. Also, stroma cells, granulosa, and follicles revealed a regular structure in the normal group. In the vitrified group compared to the control group, tissue cohesion was less, and there were more primordial follicles than other follicle types. In this group, the order of granulosa cells and the structure of follicles at different stages of growth (except for primordial follicles) were disrupted. Also, the number of primary and secondary follicles in vitrified + 1 µM kisspeptin and vitrified + 10 µM kisspeptin groups was more than in the vitrification group. In the secondary follicles, the surrounding granulosa cells and the antrum cavity were visible. In the vitrified + 1 µM kisspeptin group and the vitrified + 10 µM kisspeptin group, the number of primordial follicles was reduced. In most follicles, the nuclei were damaged and not seen due to the staining processes (Figure 2).

### Gene expression

#### 1- GDF9

**Figure 3:**
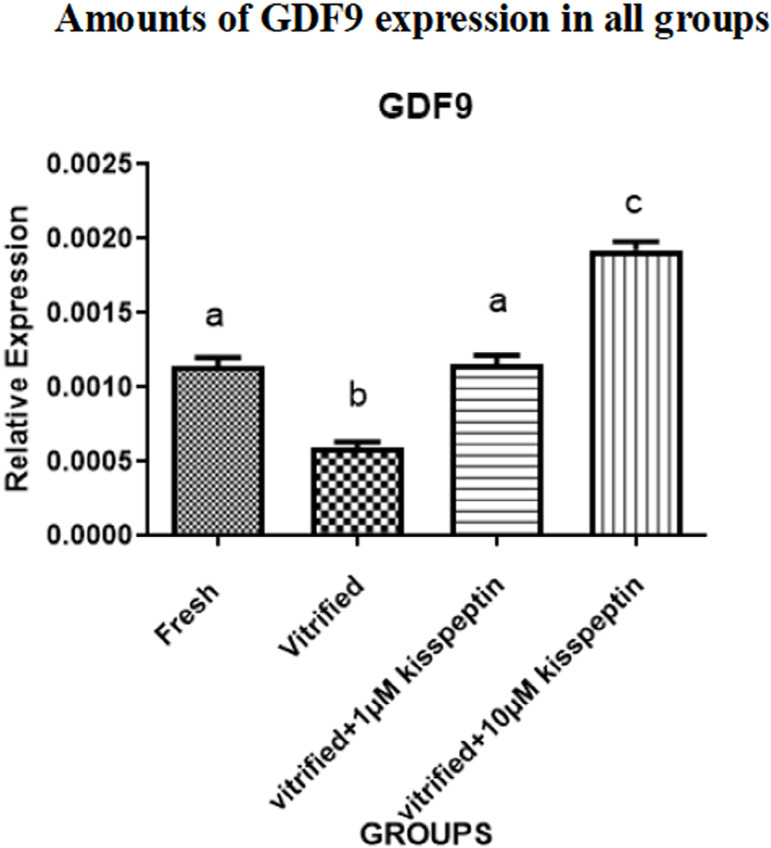
Expression of GDF9 gene in different groups after seven days of the vitrification and seven days of culture in DMEM medium. Data are shown as Means ± SD (One way ANOVA, Tukey´s test, *p*<0.05).

In the vitrification group, the expression of the *GDF9* gene was significantly reduced in comparison with other groups (*p*<0.05). Plus, the expression of this gene was elevated in the vitrified + 1 µM kisspeptin and vitrified + 10 µM kisspeptin groups than in the vitrified groups (*p*<0.05). In addition, gene expression of *GDF9* in the vitrified + 10 µM kisspeptin group was higher than in the vitrified + 1 µM kisspeptin group (*p*<0.05). Also, there were no significant differences in the expression of the *GDF9* gene between the vitrification + 1 µM kisspeptin and the control groups (*p*<0.05) (Figure 3).

#### 2- BMP15

**Figure 4:**
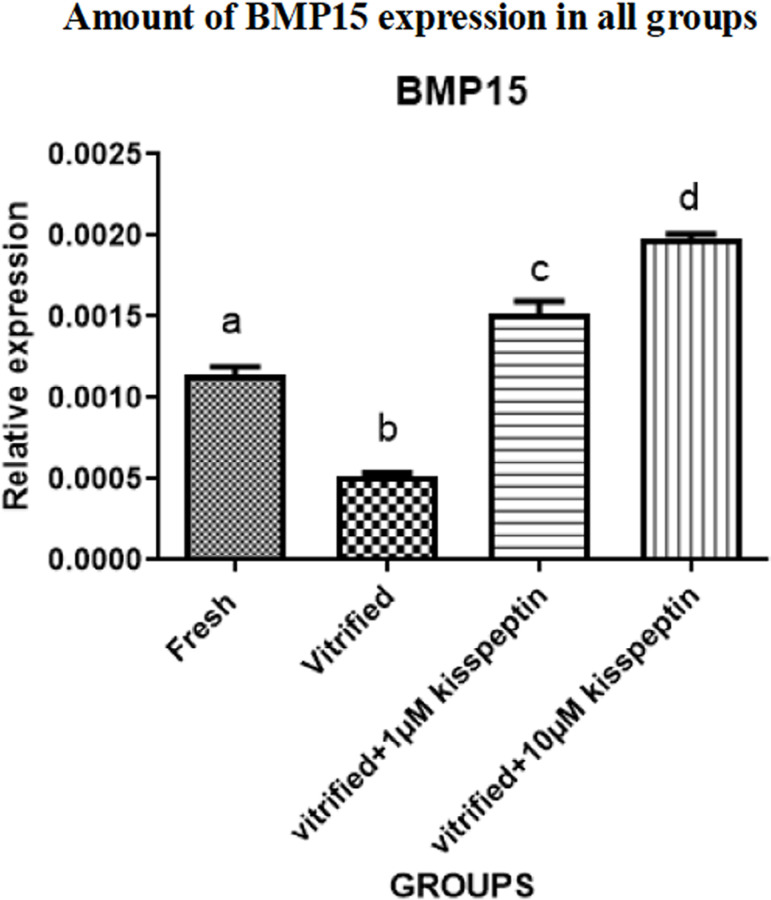
BMP15 gene expression after seven days of the glass freezing and seven days of culture in DMEM medium. Data are shown as Means ± SD (One way ANOVA, Tukey´s test, *p*<0.05).

*BMP15* gene expression was significantly decreased in the vitrified group compared with other groups (*p*<0.05). The expression of this gene was also elevated in the vitrified + 1 µM kisspeptin and vitrified + 10 µM kisspeptin groups compared with the fresh and vitrified groups (*p*<0.05). In addition, gene expression of *BMP15* in the vitrified + 10 µM kisspeptin group was higher than in the vitrified + 1 µM kisspeptin group (*p*<0.05) (Figure 4).

### Protein expression

#### 3- GDF9

The protein expression of the *GDF9* gene was dramatically diminished in the vitrified group compared with other groups (*p*<0.05). The protein expression of the mentioned gene in the vitrified + 1 µM kisspeptin and vitrified + 10 µM kisspeptin groups was significantly increased than in the vitrified group (*p*<0.05). Also, the protein expression of *GDF9* in the vitrified + 10 µM kisspeptin group was higher than in the vitrified + 1 µM kisspeptin group (*p*<0.05) (Figures 5A and B).

**Figure 5A:**
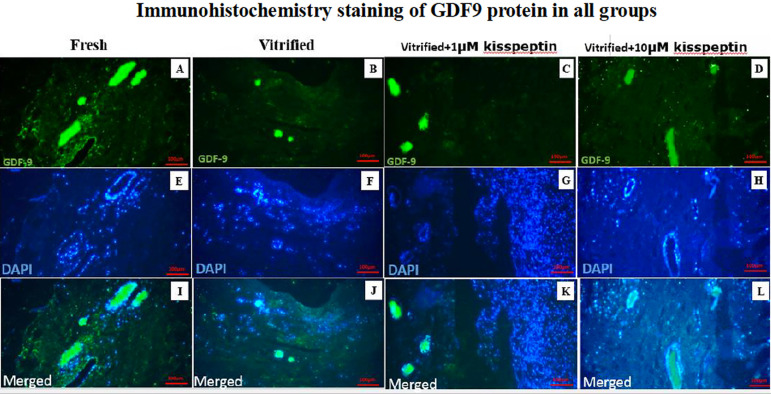
In A, B, C, and D, the green fluorescent color indicates the expression of GDF9 protein in the ovarian tissue in different groups after seven days of the vitrification and then seven days of culture in DMEM medium. In E, F, G, and H, the cell nuclei turned blue. In I, J, K, and L, the combination of the two above rows can be seen.

**Figure 5B:**
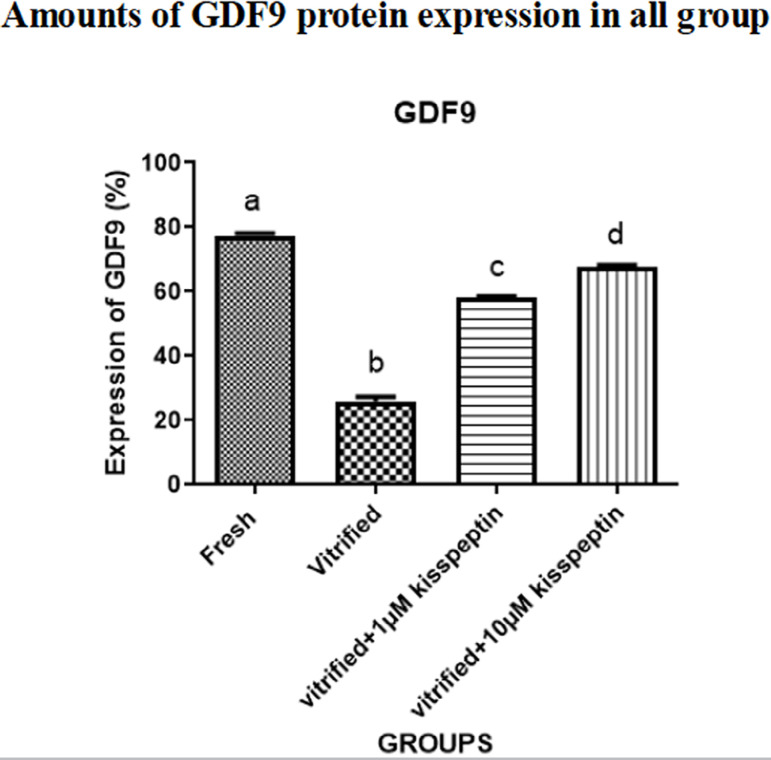
evaluation of the average percentage of gdf9 protein expression in different groups after seven days of the vitrification and seven days of culture in dmem medium. data are shown as means ± SD (One way ANOVA, Tukey’s test, *p*<0.05).

#### 4- BMP15

The protein expression of the *BMP15* gene was significantly decreased in the vitrified group than in other groups (*p*<0.05). The protein expression of this gene was dramatically elevated in the vitrified + 1 µM kisspeptin and vitrified + 10 µM kisspeptin groups compared with the vitrified group (*p*<0.05). Plus, the protein expression of *BMP15* in the vitrified + 10 µM kisspeptin group was higher than in the vitrified + 1 µM kisspeptin group (*p*<0.05) (Figures 6A and B).

**Figure 6A:**
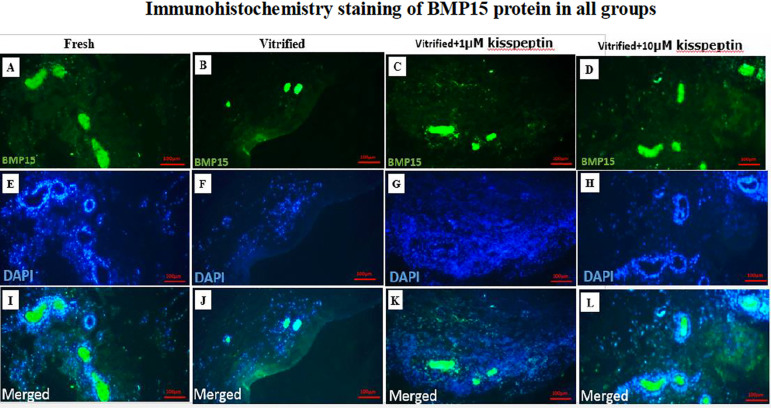
In A, B, C, and D, the green fluorescent color indicates the expression of BMP15 protein in the ovarian tissue in different groups. In E, F, G, and H, the nuclei of normal cells turned blue. Also, in I, J, K, and L, the combination of the first and second rows can be seen.

**Figure 6B:**
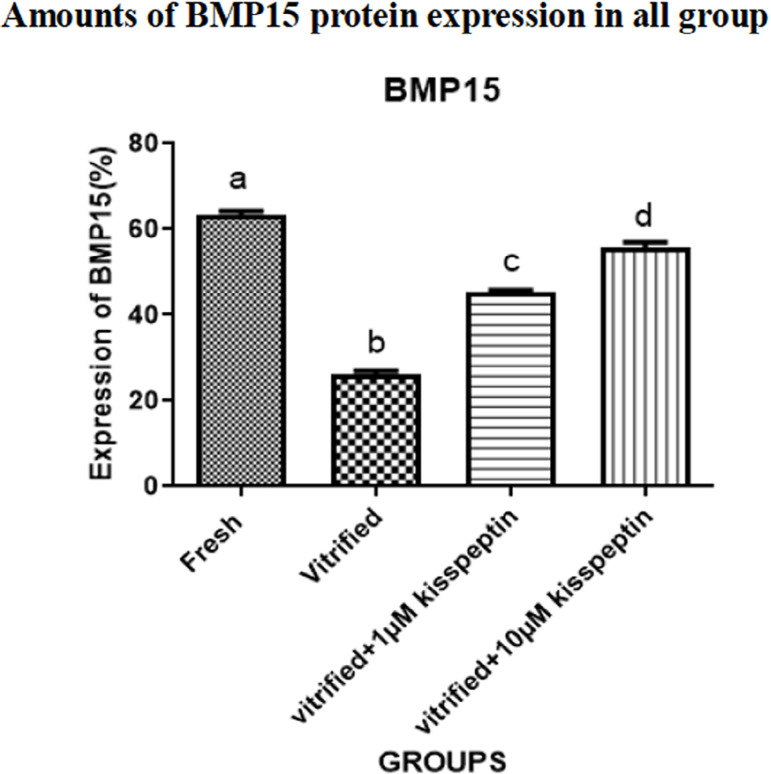
Evaluation of the average percentage of BMP15 protein expression in different groups after seven days of the vitrification and then seven days of culture in DMEM medium. Data are shown as Means ± SD (One way ANOVA, Tukey´s test, *p*<0.05).

## DISCUSSION

Ovarian tissue cryopreservation through vitrification and thawing techniques is a potential tool to preserve fertility in women who suffer from cancer. However, this method is accompanied by ROS production and subsequently DNA damage, which can have detrimental effects on the oocytes and ovarian follicles (Tsai-Turton & Luderer, 2006; Silva *et al*., 2018; Tamura *et al*., 2020). The optimization of this approach has not been carried out yet (Nyachieo *et al*., 2013; Dolmans & Donnez, 2021).

On the other hand, using antioxidants has been recommended as a practical way to minimize the production of ROS during cryopreservation (Silva *et al*., 2018). In this regard, we evaluated the effectiveness of kisspeptin, as an antioxidant agent (Aslan *et al*., 2017), on follicle maturation and the expression of relevant genes and proteins (*BMP15* and *GDF9*) based on ovarian tissue culture following vitrification-thawing processes.

In this study, the histological results revealed a reduction in the number of follicles in all growth stages with the exception of primordial follicles in the vitrification group than in the fresh group. These findings may reflect the negative effects of the vitrification on follicle maturation in light of ROS formation (Chang *et al*., 2019; Shoorei *et al*., 2019). Similarly, Migishima *et al*. (2003) indicated a decrease in the number of follicles in frozen-thawed ovaries than in fresh ovaries. However, some documents reported normal morphology of ovarian follicles after the vitrification (Salehnia *et al*., 2002; Luvoni *et al*., 2012). We also observed that the number of primary and secondary follicles in vitrification groups with kisspeptin (1 and 10 µM) was more than in the vitrification group.

It has been declared that kisspeptin potentiates the induction of ovarian follicle maturation and estradiol production by promoting FSH synthesis (Hagen *et al*., 2014; Rather *et al*., 2016). The line with this notion, some published papers emphasized the role of this antioxidant in improving follicle development and maturity (Taniguchi *et al*., 2017; Magamage *et al*., 2021). In this regard, a study revealed that the administration of kisspeptin increases the number of preovulatory follicles and raises the plasma AMH secreted by secondary follicles (Cao *et al*., 2019). In this study, the gene and protein expression of *GDF9* and *BMP15*, as follicle maturity agents, in the vitrification group was significantly reduced compared with the control group. Consistent with this finding, Choi *et al*. (2007) showed diminished expression of some markers related to follicle development, like *GDF9*, after cryopreservation of mouse ovaries. Also, Ebrahimi *et al*. (2010) reported that the expression of *GDF9* and *BMP15* was decreased owing to freezing sheep oocytes. Unlike these studies, some researches manifested that the gene expression of *GDF9* did not have a significant difference compared to the control group after freezing (Ramezani *et al*., 2017). These differences may be related to differences in cryopreservation methods (Stachowiak *et al*., 2009). Another report was that the gene and protein expression of *GDF9* and *BMP15* in the vitrification groups with kisspeptin was significantly elevated compared with the vitrification group. In corresponding with this result, Saadeldin *et al*. (2012) demonstrated increased expression of *GDF9* and *BMP15* genes in the time of in vitro maturation of oocytes after kisspeptin administration. Also, it is stated that oocyte maturation stimulated by kisspeptin is in light of *GDF9* and *BMP15* upregulation (Hu *et al*., 2018). *BMP15* and *GDF9* are crucial growth factors for regulating luteinization, folliculogenesis, ovulation, developmental competency, and oocyte maturation (Hu *et al*., 2018). In addition, their proteins are expressed in the primary stage of follicles and play an important role in the transfer of follicles from this stage to the secondary (Kona *et al*., 2016; Celik *et al*., 2020).

It looks like kisspeptin can serve as an obstacle against the destructive effects of the vitrification on follicle growth and maturation by elevating antioxidant defense processes and upregulating follicle maturity genes, like *GDF9* and *BMP15*.

### Limitation

In this study, due to the observance of ethical protocols, the number of samples was small, and less than 10% of the patient’s tissue was removed, so we were not able to perform further tests.

## CONCLUSION

Our findings indicated that kisspeptin is a suitable substance to potentiate the quality of the human ovarian cryopreservation medium. Indeed, kisspeptin can increase the maturity of follicles and is effective to increase the possibility of a successful pregnancy using assisted reproductive techniques in clinics.

## ABBREVIATIONS

POI: Primary ovarian insufficiency

OTC: Ovarian tissue cryopreservation

ROS: Reactive oxygen species

HAMs: Ham’s tissue culture medium

DMSO: Dimethyl sulphoxide

HEPES: 4-(2-hydroxyethyl)- 1-piperazineethanesulfonic acid

ES: Equilibration solution
VS: Vitrification solution
DMEM: Dulbecco’s modified eagle medium
FBS: Fetal bovine serum
IHC: Immunohistochemistry
DAPI: 4′,6-diamidino-2-phenylindole
GDF9: Growth differentiation factor 9
BMP15: Bone morphogenetic protein 15
FSH: Follicle stimulating hormone
LH: Luteinizing hormone
BMI: Body mass index
AMH: Anti-mullerian hormone
H&E: Hematoxylin and eosin
